# Sexual Communication in the *Drosophila* Genus

**DOI:** 10.3390/insects5020439

**Published:** 2014-06-18

**Authors:** Gwénaëlle Bontonou, Claude Wicker-Thomas

**Affiliations:** CNRS UPR 9034 and Université de Paris Sud, 91198 Gif sur Yvette, France; E-Mail: Gwenaelle.Bontonou@legs.cnrs-gif.fr

**Keywords:** *Drosophila*, courtship, pheromones, *cis*-vaccenyl acetate

## Abstract

In insects, sexual behavior depends on chemical and non-chemical cues that might play an important role in sexual isolation. In this review, we present current knowledge about sexual behavior in the *Drosophila* genus. We describe courtship and signals involved in sexual communication, with a special focus on sex pheromones. We examine the role of cuticular hydrocarbons as sex pheromones, their implication in sexual isolation, and their evolution. Finally, we discuss the roles of male cuticular non-hydrocarbon pheromones that act after mating: *cis*-vaccenyl acetate, developing on its controversial role in courtship behavior and long-chain acetyldienylacetates and triacylglycerides, which act as anti-aphrodisiacs in mated females.

## 1. *Drosophila* Courtship Behavior

While several studies have investigated the role of signals in insect communication, few reviews present all the factors involved in courtship behavior. However, these factors can act in a combined manner to allow mating and be involved in the establishment of sexual isolation. In this paper, we review current knowledge about signals involved in sexual communication in *Drosophila*, with special focus on chemical signals. We describe *Drosophila* cuticular hydrocarbons (CHCs), examine their role as sex pheromones, their role in sexual isolation, and their evolution. Finally, we provide a critical survey of the literature concerning an important non-hydrocarbon pheromone: *cis*-vaccenyl acetate.

**Figure 1:**
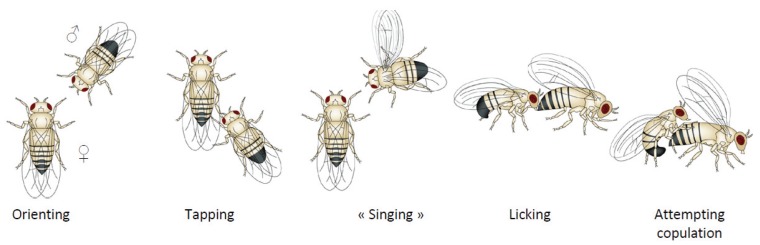
Sequence of sexual courtship ritual in *Drosophila* (adapted from [9]).

The *Drosophila* genus is composed of a wide range of species, some of which are genetically close, living in different environments in allopatry or sympatry. Their reproductive behavior is diverse in terms of sperm utilization, capacity for re-mating, or settling of courting [1]. Courtship behavior was first described in *Drosophila melanogaster* in 1915 [2] and later in other species of the *Drosophila* genus. The courtship ritual is composed of stereotyped but nonlinear steps that constitute a true dialog between the partners [3,4,5]. Courtship choreography, illustrated in Figure 1, is as follows: when a male encounters a potential female mate, he orients toward her and taps her abdomen with one of his front legs. This allows him to perceive her pheromonal characteristics. If he decides to pursue courtship, he follows her, extends the wing that is closest to her and produces a “love song” [6,7]. He also vibrates his abdomen, creating substrate-born vibrations that are transmitted to the female [8]. He will then lick her genitalia with his proboscis and attempt to copulate by bending his abdomen.

Depending on female receptivity, some or all these steps can be repeated until copulation occurs. The female transmits acceptance or rejection signals to the male throughout courtship [2,10]. A receptive female will reduce her locomotor activity and partially extrude her ovipositor and some emit a droplet, which is excitatory to the male [11]. Conversely, a non-receptive female will kick the male, move her abdomen up and down, run away, totally extrude her ovipositor, and will keep her wings closed preventing the male from positioning [3,11]. Mating leads to physiological and behavioral modifications in both partners, which will temporarily alter their attractiveness, reducing their ability to re-mate. These modifications have been extensively studied in females [12,13,14,15], but less so in males [16]. Signals emitted during courtship have characteristics, which are to a species specific or unique to a given population and are important for intra-specific recognition and reproductive success.

## 2. Non-Chemical Stimuli

### 2.1. Acoustic Stimuli

**Figure 2:**
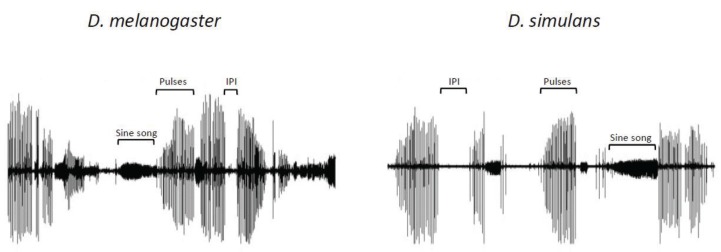
Courtship song of *Drosophila melanogaster* and *Drosophila simulans* males recorded for 10 s. The original figure (from [23]) has been modified for this review.

During courtship, males of most *Drosophila* species emit sounds (songs) by vibrating one or both wings. Courtship songs are very diverse in the genus *Drosophila* and their acoustics characteristics are specific to species in the *melanogaster* sub-group [17]. They could contribute to reproductive isolation in these species [18]. *D. melanogaster* males produce two songs: a series of rattles (pulse song), produced by short wing vibrations and bursts of humming (sine song), produced by longer wing vibrations (Figure 2) [19,20]. Pulse song is present in all species that produce sounds and in those that emit two or three songs pulse song is associated with sinusoid song. Inter-pulse interval (IPI) duration and frequency are highly variable between species of the *melanogaster* sub-group [17]. For example, *Drosophila simulans* and *D. melanogaster* songs differ in pulse frequency (480 Hz *vs.* 280 Hz) and IPI length (55 ms *vs.* 30 ms) [17,21]. Within species these parameters also vary between males and those with a greater variability in IPI seem to stimulate more females [21,22,23].

In many *Drosophila* species, wing song has an important role in inciting females to mate [7,24,25] and how quickly the females become receptive to the male [26]. The pulse song and in particular IPI are critical parameters for inter-species recognition [27,28,29,30]. The importance of IPI in sexual isolation of closely related species living in sympatry has been evidenced. Some species are able to mate only when they hear songs specific to their species [31,32,33,34,35], while others rely on the presence of heterospecific songs to discriminate their partner and eventually mate [29,36,37]. In some species however, song has only a secondary role in courtship specificity [38]. The role of courtship song in selecting a conspecific partner is not fully understood, as little variability is observed between males from the same species [39]. Nevertheless, it has been shown that *D. melanogaster* females favor males with long pulse song and *Drosophila montana* females prefer songs with short but frequent pulses [40]. Producing pulses is very energy demanding and might give to the female an indication of male fitness [41].

A female can also emit sounds before courtship, which help the male to locate her [42]. During courtship, females, except those of the *virilis* group [43,44], generally do not produce specific songs [18]. However females can flick their wings as a mark of rejection when they hear a non-specific song. In *Drosophila ananassae* and *Drosophila pallidosa*, two genetically close species, females perform wing vibrations in response to a heterospecific male song, which results in the arrest of the courtship. This in itself is responsible for sexual isolation between these two species [45]. It should be noted that wing vibration plays a role in different steps of courtship by transmitting acoustic and visual information to the partner and also by dispersing pheromones, which are normally perceived at close proximity. 

### 2.2. Visual Stimuli

Visual stimuli can be dynamic (movements, locomotion) or static (pigmentation, color, form) and vary quantitatively and qualitatively within the *Drosophila* genus. Dynamic signals intervene at different steps during courtship and allow the male to detect the presence of another individual that is moving. The male will orient towards an individual, and, if it appears to be female, he will touch it with his anterior leg [46]. The movements and locomotor activity of the female will then inform the male about her receptivity. The male, thus, needs his vision [47] and more particularly his capacity to detect movements [26,48] to allow him to stay in proximity of the female during the first steps of courtship, a period in which the female is more likely to escape. In some *Drosophila* species, static signals can also modulate female choice. In the Hawaiian drosophilidae, the female is influenced by the pattern present on the males’ wings, explaining the great diversity of wing patterns in these species [49]. In *Drosophila suzukii*, mating frequencies of males with or without wing patterns are identical in darkness, whereas females prefer males with spotted wings in the light [50]. The importance of visual stimuli in the initiation and success of courtship is highly variable between species [51,52]. Some species, such as *Drosophila auraria*, do not mate or infrequently mate in the dark. Others, such as *D. melanogaster*, mate indifferently in the presence or absence of light. In the latter species, other sexual stimuli or factors linked to environment (such as food) are likely to play a major role in the settling of courtship. A comparison between the expression of *D. melanogaster* and *D. simulans* genes by micro-array has shown that *D. simulans* males overexpress genes involved in phototransduction, confirming that visual signals are more important for mating in *D. simulans* than in *D. melanogaster* [53]. In *D. melanogaster* males, two genes related to olfaction are upregulated, highlighting the importance of pheromonal cues in this species.

## 3. Sex Pheromone Signals

### 3.1. Cuticular Hydrocarbons-Generalities

**Figure 3:**
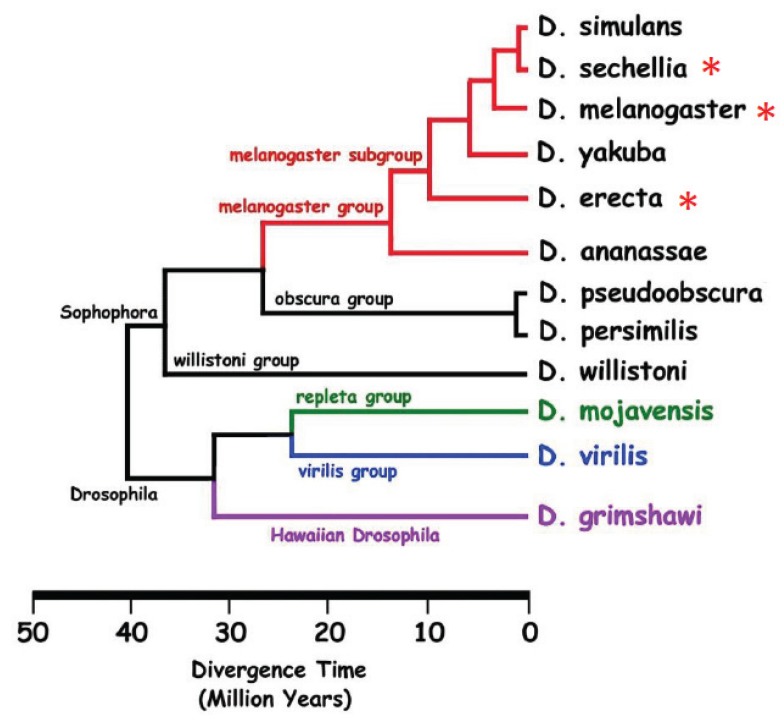
Phylogenetic relationship between different *Drosophila* species cited in the text. Red asterisks show the species that present qualitative sex cuticular hydrocarbon polymorphism.

**Figure 4:**
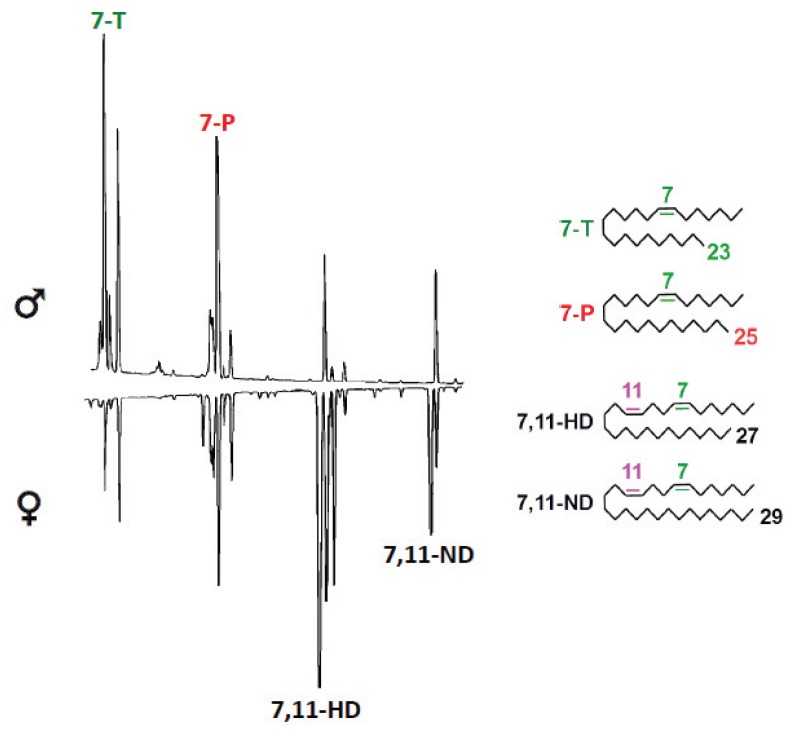
Typical chromatograms of cuticular hydrocarbons from a male and female cosmopolitan *D. melanogaster* strain (Canton-S). The main pheromones are indicated on the chromatogram on the left and their chemical structures are shown on the right.

In the *Drosophila* group, cuticular hydrocarbons (CHCs) act as recognition signals and sex pheromones. They are perceived at a short distance by olfactory organs on the head (antennae and maxillary palps) and/or by contact with the tarsi and proboscis gustative organs [54,55,56]. There are fewer CHCs compared to other insects [57], however, there is a high diversity concerning chain length, the number and position of unsaturations and the existence, in some species, of sex dimorphism and intra-specific variation. We will focus here on a limited number of species, the phylogenetics of which are represented in Figure 3. CHC length can vary from 20 to 40 carbons. Some species have a larger synthesis spectrum than others and therefore a wilder cuticular signature. *Drosophila birchii*, for example, synthesizes CHCs ranging from 20 to 33 carbons, whereas *Drosophila peniculipedis* (Hawaiian species) males have only 25 and 27 carbon CHCs [58]. Chain length is well conserved within groups: Hawaiian drosophilae and drosophilae from the *Sophophora* sub-group produce the shortest CHCs (between 23 and 29 carbons in most species, but up to 33 carbons in *D. ananassae* and *Drosophila erecta*); the *virilis* group species produce CHCs that are slightly longer (between 22 and 31 carbons); and the *repleta* group species synthesize the longest (between 28 and 40 carbons). The compounds that constitute *Drosophila* hydrocarbon profiles belong to a limited number of classes. They are generally *n*-alkanes, unsaturated compounds with one or several double bonds (monoenes, alkadienes, alkatrienes) and alkanes with a methyl group. Some species, such as *D. melanogaster*, synthesize all or most of these compounds whereas others such as *Drosophila willistoni* and *Drosophila elegans* synthesize a few of them. Interspecific differences can be qualitative and also quantitative. The highest variation, particularly in those involving CHC sex dimorphism, occurs in the *melanogaster* sub-group. Male hydrocarbon profiles are extremely similar: in all species, almost of the half of CHCs are 7-tricosene (C23:1; 7-T) and, with the exception of *Drosophila erecta* males, 7-pentacosene (C25:1; 7-P) (Figure 4) [59,60,61].

Hydrocarbon profiles of females, contrary to those of males, show more variability between species. *D. melanogaster*, *Drosophila sechellia*, and *D. erecta* females represent a sex hydrocarbon dimorphism, and produce longer CHCs, less monoenes, and more dienes that act as pheromones. The other species within the group have qualitatively the same CHCs as their conspecific males, but have different concentrations [61,62]. Several CHCs (*n-*alkanes and branched alkanes) are present at a low abundance but similar amounts in males and females of these species [63], and might correspond to ancestral hydrocarbons.

### 3.2. Pheromonal Role of CHCs

In *Drosophila*, some CHCs, generally the most abundant, act as sex pheromones during courtship behavior and mating [59]. The pheromone bouquet is composed of several stimulatory or inhibitory compounds that can be differently perceived between populations. In *D. melanogaster*, male 7-T can both inhibit homosexual courtship and stimulate the females of some populations [64,65,66]. Conversely, in *D. simulans*, 7-T is generally more abundant in females and stimulates conspecific males. The high variability in CHC numbers and their amounts could serve to improve attractiveness of potential mates from the same species or population and could be directly involved in assuring intraspecific matings and in reproductive isolation between species [67,68,69,70,71,72].

The importance of female sex pheromones in the pre-reproductive barrier is well established in the *melanogaster* group. Males belonging to sex monomorphic CHC species do not generally court females from sex dimorphic CHC species, whereas males from sex dimorphic CHC species infrequently court females from sex monomorphic CHC species. The main pheromone of *D. melanogaster* and *D. sechellia* females, 7,11-heptacosadiene (C27:2; 7,11-HD; Figure 4), is responsible for these behaviors [73]. *D. melanogaster/D. simulans* hybrid females produced pheromone dienes and were rarely courted by *D. simulans* males and never mated, whereas hybrids with a chromosomal deletion of the *D. melanogaster desatF* gene had no dienes and received normal courtship from *D. simulans* males and eventually mated [74]*.*

Flies devoid of CHCs can be generated by genetic ablation of oenocytes [75]. Surprisingly, females lacking CHCs appeared more attractive than those with a normal CHC profile: *D. simulans* and *D. yakuba* males do not court *D. melanogaster* females but court those devoid of CHCs. Treatment of these females with 7,11-HD can restore the species barrier: the addition of synthetic 7,11-HD to *D. melanogaster* females without CHCs is sufficient to totally suppress courtship behavior from *D. simulans* and *D. yakuba* males toward these females [75]. Likewise, the addition of synthetic 7,11-HD to wild-type *D. simulans* or *D. yakuba* females is sufficient to inhibit courtship behavior from conspecific males but will stimulate *D. melanogaster* males [76]. One case, from the *melanogaster* sub-group suggests that male pheromones might be responsible for reproductive isolation: *D. santomea* and *D. yakuba*, two sister species inhabiting Saõ Tomé island (Gulf of Guinea), display strong reproductive isolation [77]. Their CHC profile is similar, with the exception of one compound, *n*-heneicosane (C21:0), which is up to seven times more abundant in *D. santomea* males than in *D. yakuba*. The females of both species discriminate this CHC, which is attractive to *D. santomea* and repellant to *D. yakuba* [78].

### 3.3. Pheromonal Role of CHCs from Immature Flies

Homosexual behavior has been described in *Drosophila*. Mature *D. melanogaster* males vigorously court immature males [79,80]. In fact, immature flies contain complex CHCs mixture with 27 to 37 carbons, most of them methyl-branched or mono- or di-unsaturated [81]. These particular unsaturated CHCs could explain the attractiveness of young flies—males and females—to mature males. Once a male has been rejected by an immature fly, he will perform much less courtship to immature flies [82] and that will allow him to maximize the time he devotes to courting sexually receptive females [83]. Mature males rarely court other mature males, due to the repellent effects of 7-T and cVA ([64]; Section 6.2). 

## 4. Role of CHCs in Reproductive Isolation

### 4.1. Role of Female Pheromones in D. melanogaster Reproductive Isolation

CHC profiles can vary within species and more and more studies report non-random mating between divergent populations. Whilst several studies have been done in *D. elegans* [84] and *D. montana* [85], cases of intra-specific sex isolation are best documented in *D. melanogaster*. In this species, a CHC polymorphism linked to the geographic origin of the population results in the production of different female sex pheromones. Females from most populations, named “cosmopolitan populations”, produce high amounts of 7,11-HD, whereas females originating from West-Africa, Zimbabwe, and the Caribbean, synthesize little 7,11-HD but large quantities of a position isomer, 5,9-heptacosadiene (Figure 4) [86]. Reproductive isolation has been described between African populations from Zimbabwe and populations from America [87], and also between populations from Sub-Saharan Africa and the Caribbean and populations from other continents [88]. These extremely differentiated populations represent a model of speciation at the nascent stage [89]. The gene involved in this female pheromone polymorphism has been characterized, nevertheless, its exact role in reproductive isolation has yet to be clarified [90,91,92,93].

### 4.2. Role of Male Pheromones in D. melanogaster Reproductive Isolation

In *D. melanogaster*, two studies suggest that male pheromones play a role in reproductive isolation between populations. The first study concerns the Zimbabwean population. Populations from the Caribbean mate at high frequency with those of West-Africa and *vice versa* but both are sexually isolated from Zimbabwean populations, even though females have similar CHC profiles. Other parameters involving the male, such as morphology, courtship behavior or pheromones might also be responsible for this isolation. A recent study has shown that Zimbabwean females could discriminate their own males from others, due to the presence of high 5-T in Zimbabwe males [94].

The second study concerns the populations where males produce “high”7-T or “low”7-T [95]. There is a geographic CHC polymorphism concerning the males: Cosmopolitan males synthesize large quantities of 7-T (and low quantities of 7-P: phenotype “7-T”), contrary to males living in hotter areas, which have high and low quantities of 7-P and 7-T, respectively (phenotype “7-P”) [96]. This polymorphism confers a superior resistance of 7-P males to hot conditions [97]. Although populations from different locations have clear-cut CHC phenotypes (either 7-T or 7-P), we found a population from the Comoro Islands, in which males fell on a continuum ranging from low to high levels of 7-T. After 15 generations of selection for CHC profile, the “7-T” and “7-P” selected lines showed reproductive isolation [95]. Likewise, other experiments conducted on 7-T and 7-P strains of *D. simulans* show reproductive isolation between strains with different CHC phenotype (7-T or 7-P) suggesting that male CHCs could contribute to sex isolation in this species [98]. Several factors involved in courtship behavior or female receptivity could also contribute to sexual isolation. At least three loci controlling female mating preference and four loci responsible for male mating success have been localized [99], suggesting that numerous genes could co-evolve.

## 5. Plasticity and Evolution of CHCs

### 5.1. Plasticity of Hydrocarbons

Several factors may affect hydrocarbon profiles, notably temperature, food, and social context [100,101,102,103]. The impact of temperature will be discussed here. In *D. melanogaster*, *D. mojavensis*, *D. pseudoobscura*, and *D. serrata*, selection for resistance to desiccation leads to a modification of hydrocarbon profiles, which is generally characterized by an increase in chain length [104,105,106]. Long chain CHCs have higher melting points which gives them a superior capacity to limit water loss compared to short chain CHCs [107]. The main pheromones in *D. melanogaster* males, 7-T and 7-P, could therefore be involved in heat and desiccation resistance. A study in 85 *D. melanogaster* populations has shown that the ratio 7-T/7-P significantly varies with latitude, mean temperature range and water pressure [96]. Flies reared at 29 °C have more 7-P and less 7-T than flies reared at 18 °C [97].

### 5.2. Evolution of CHC Profiles

Natural and sexual selection can play a role in CHC evolution. When close species or isolated populations come into geographical contact, hybrid matings may occur and give non-viable or sterile offspring. To prevent maladaptive hybridization, natural selection enhances pre-zygotic isolation between these species or populations. This process is called reinforcement. Key studies looking at the impact of these evolutionary forces and their consequences on the establishment of sex isolation have been done in *D. serrata*. *D. serrata* and *D. birchii* are two Australian species which have different but overlapping distributions. *D. serrata* populations that live in sympatry with *D. birchii* display CHC profiles vastly different from those of populations living in allopatry. When natural sympatric and allopatric populations of *D. serrata* were exposed to experimental sympatry with *D. birchii* for nine generations, CHC profiles of allopatric *D. serrata* populations evolved to resemble the sympatric populations, whereas sympatric populations remained unchanged [72]. Complementary studies have also shown that both sexes of *D. serrata* choose partners according to their CHCs but these choices are made in an opposing way: males prefer females with intermediary profiles, resulting in stable sexual selection for female CHCs [108], whereas females exert a strong and unidirectional selection on male CHC profiles, which is responsible for their evolution [109,110]. Allopatric females prefer males with an allopatric CHC profile [111], resulting in a selection against sympatric profiles in areas where different populations co-exist. This results in an absence of gene flow between different populations [110]. In sympatric populations, reproductive character displacement by reinforcement compromises sexual selection. Reinforcement and sexual selection lead to populations with different CHC profiles and altered preferences for these CHCs, which can favor the establishment of a reproductive isolation.

Natural selection could also strongly impact the CHC evolution of different populations. As previously mentioned, insects living in hot environments synthesize longer chain CHCs. Experimental populations subjected to different environmental conditions can produce different CHCs and as a result, females modify their preferences for male CHCs [112]. The divergences of CHC preferences that result from an adaptation to a new environment could be an important cause of reproductive isolation. Several studies have shown that natural and sexual selection can influence CHC evolution and male reproductive success. However, sexual selection is unlikely to cause divergence among natural populations without a concomitant switch in natural selection [113].

In other species, it is less clear how CHCs evolve. One study suggests that in *D. melanogaster* the female CHC polymorphism might have been originated from an adaptation to colder conditions [114]. However, these results could not be reproduced [93]. The link between CHC evolution in different environmental conditions and the evolution of sexual preference is not clearly established [105,112,115]. A recent study suggests that methyl-branched CHCs could affect mate choice and desiccation resistance: *D. serrata* and *D. birchii* show prezygotic isolation. The former species produces methyl-branched CHCs, contrary to the latter species and is also more resistant to desiccation. This class of CHCs could be important in the adaptation of *Drosophila* to different environments and in reproductive isolation [116].

The recent isolation of a *D. melanogaster* population where males have a divergent CHC profile provokes thought on whether the presence of different CHCs in the same environment is also of significance [95]. Our experiments suggest that these flies share a common genetic background and a strong correlation was found between 7-P content and resistance to desiccation. Moreover, when these flies were maintained for two years at different temperatures (21 and 25 °C), the flies reared at 21 °C evolved to a 7-T predominant phenotype and the flies reared at 25 °C to a 7-P predominant phenotype, suggesting that CHC profile might be determined by temperature conditions. The male polymorphism could provide an advantage in highly changing environmental conditions.

## 6. Non-Hydrocarbon Pheromones

### 6.1. cis-Vaccenyl Acetate

#### 6.1.1. Production of *cis*-Vaccenyl Acetate

**Figure 5:**
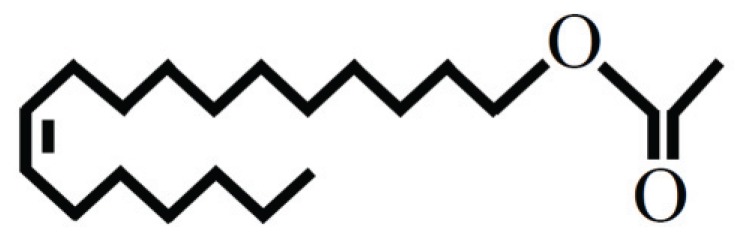
Molecular structure of *cis*-vaccenyl acetate.

All species of the *D. melanogaster* group produce *cis*-vaccenyl acetate (cVA; (*Z*)-11-octadecenyl acetate; Figure 5) [117,118]. This ester is exclusively synthesized in the male ejaculatory bulb [119]. It can be extracted together with cuticular hydrocarbons using hexane wash and is present in quantities of approximately 200 ng in male Canton-S [120]. cVA is considered male-specific; however, trace amounts of cVA were found on the cuticle of virgin females, using a method based on direct ultraviolet laser desorption/ionization orthogonal time-of-flight mass spectrometry (UV-LDI-o-TOF MS) [121].

In *D. melanogaster* species, the amount of cVA varies between 88 and 230 ng, depending on the strain [122]. cVA amounts increase with age [116] and are also dependent upon rearing temperature [123].

A small quantity of cVA is found on the integument of the male. However, it is difficult to evaluate the exact amount released onto the cuticle because solvent extraction may remove also some cVA derived from inside the fly. A 1 min extraction would be in principle sufficient to recover the cuticular cVA, whereas longer extractions may recover cVA associated with the entire fly. With a 1 min extraction, the proportion of cVA on the tegument was estimated at about 50 ng in mature males [124].

#### 6.1.2. Role of cVA in Mating Behavior

A significant amount of cVA present in the male is transferred to the female during mating along with the seminal fluid. Jallon *et al.* [120] estimated that 185 ng was recovered in a female two hours after copulation. Most of this cVA is present in the reproductive tract and only 20 ng is found on the tegument just after mating [124]. After mating, a waxy plug, formed by ejaculatory bulb secretions, appears inside the uterus. This causes the uterus to dilate, thus, helping the sperm to reach the storage organs [125].

cVA has been thought to act as an anti-aphrodisiac pheromone, preventing males mating newly inseminated females but also interactions between males. Topical application of 50 ng cVA to virgin females leads to a 60% inhibition in male courtship [120,126,127,128]. However, most of the cVA transferred to the female during mating is deposited in the uterus and it is unknown what proportion of cVA leaks from the uterus to the female cuticle. It is likely that this secretion is limited, suggesting that cVA may play a very limited role—if any—in repelling males after copulation [129]. Several studies have refuted the anti-aphrodisiac role of cVA in mated females [64,122,129]. More recently, Billeter *et al.* observed that perfuming females devoid of CHCs with cVA delayed mating but this delay was eliminated when these females were treated with a blend of cVA and 7,11-HD [75]. The decreased attractiveness of mated females can also be due to other CHCs transferred to the female’s cuticle during mating: 7-T, rubbed off onto *D. melanogaster* females during mating, is perceived as an antiaphrodisiac pheromone by males [64].

#### 6.1.3. Role of cVA as an Aggregation Pheromone

The cVA transferred to the female during mating is deposited onto the rearing medium within 6 h after mating completion and attracts flies of both sexes [117]. The mating sites are particularly attractive to virgin females, which assemble and remain at food sources, where mating had taken place [130]. About 1 μg of cVA is needed for this aggregation effect [117]. It acts in synergy with food odors (ketones, aldehydes) to attract other flies [117,131].

cVA is present in all the species of the *Sophophora* subgenus and in several species of the *Drosophila* subgenus, for example in *D. immigrans* [132]. In other species other compounds, which are more or less structurally related to cVA, can act as aggregation pheromones: (*Z*)-11-hexadecenyl-acetate in the *immigrans* group, (*Z*)-11-hexadecenyl-acetate and (*Z*)-11-eicosenyl-acetate in the *D. quinaria* group [132,133]. The distribution of aggregation pheromones among species is congruent with their phylogeny and an increase in the structure difference is correlated with phylogenetic distance [134]. The lack of species specificity pheromones constitutes a benefit for flies allowing them to share an oviposition/egg-laying substrate. This leads to an enhancement in survival and growth of offspring [135]. However, when fly density is too high, female oviposition is reduced and competition for food among larvae may occur [136].

#### 6.1.4. Other Behavioral Roles of cVA

When population density is low, cVA is present at a relatively low concentration and acts as an aggregation pheromone, resulting in increased number of flies on the same food source. cVA concentration increases with the number of male flies present. High quantities of cVA (500 μg) lead to male-male aggression and their dispersal from the food source [137]. This highlights a possible role of cVA in controlling population density. Contrary to acute cVA exposure which elicits male-male aggression, chronic exposure reduces aggression [138]. Surprisingly, these different effects of cVA on the same behavior are mediated by two different types of neurons. This could mimic the effect of group housing and contribute to social modulation of aggressiveness.

### 6.2. Polar Compounds and Triacylglycerides

Recently, the use of non-conventional analytical methods has allowed the characterization of more polar cuticular compounds. Direct UltraViolet Laser Desorption/Ionization Mass Spectrometry (UV-LDI MS) has been used to analyze cuticular compounds, many of which are not detected by GC/MS [121]. One of these compounds is a male-specific sex pheromone named CH503 (3-*O*-acetyl-1,3-dihydroxy-octacosa-11,19-diene). CH503 is present at high levels in the male anogenital region from *D. melanogaster* and transferred to the female during mating. Unlike cVA, CH503 remains for at least ten days after copulation on the female integument and inhibits male courtship [121].

UV-LDI MS was also used to analyze the cuticles of flies from the *Drosophila repleta* and *Drosophila quinaria* groups [103,139]. Beside long-chain acetyldienyl acetates (OAcs), triacylglycerides (TAGs) were also present exclusively on males. These sex-specific compounds, like the cVA, are synthesized in the male ejaculatory bulb [103]. The structure of these TAGs is very unusual, with at least one short-branched tiglic acid (E-2-methylbut-2-enoic acid) and one long linear fatty acid. OAcs and TAGs are secreted from the male ejaculatory bulb and transferred to females during mating. They function synergistically to inhibit courtship from other males. TAGs are broadly conserved across the subgenus *Drosophila* but absent in the subgenus *Sophophora* [103].

OAcs and TAGs represent new classes of pheromones that act, like cVA, after mating. One difficulty for their study is their unconventional structure and their diversity within species. Moreover, food might influence the amount of several TAGs [103,139]. The determination of the underlying pathways, the role of food and their behavioral function will certainly enable to understand the evolution and the role of these compounds in insect reproduction.

## 7. Conclusions

*Drosophila* is considered as a model insect that has been largely used for behavioral, genetic and molecular studies. Due to the economic importance of many dipteran species, the different signals involved in sexual communication of *Drosophila* will continue to be a focus of interest. It is now possible to introduce foreign genes into numerous *Drosophila* species, permitting to study their impact on sexual behavior. The sequencing of different *Drosophila* genomes will be helpful to better understand the genetics and the evolution of these behaviors and signals. These novel techniques will also allow to struggle against invasive species like *D. suzukii*, an economically important fruit pest introduced in America and Europe since 2008 [140].

This review also shows the importance of chemical signals, not only for reproduction purposes, but also for other behaviors, especially social ones. Still, little is known about the role of pheromones in providing social cues, however some studies suggest that olfactory cues play a role in regulating sleep, circadian rhythms [141], and on locomotor activity of socially interacting flies [142]. Social experience also affects sex pheromone synthesis and mating behavior in *Drosophila* males [143]. Future studies will have to address all these interactions.
